# Recurrent Angina in a Patient With Myocardial Infarction With Non-obstructive Coronary Arteries

**DOI:** 10.7759/cureus.37842

**Published:** 2023-04-19

**Authors:** Ngoda Manongi, Latha T Subramaniam

**Affiliations:** 1 Internal Medicine, New York-Presbyterian Queens, Queens, USA; 2 Cardiology, Nuvance Health Danbury Hospital, Danbury, USA

**Keywords:** acute st myocardial infarction, coronary artery vasospasm, myocardial infarction with non-obstructive coronary arteries (minoca), cardiac magnetic resonance (cmr), acute myocardial infarction, late gadolinium enhancement, non-st elevation myocardial infarction

## Abstract

The ubiquity of coronary angiography has increased the identification of myocardial infarction with non-obstructive coronary arteries. Currently among cardiologists, there is neither a consensus nor comprehensive diagnostic blueprint for accurate evaluation of patients with myocardial infarction with non-obstructive coronary arteries. We present a case of a patient with recurrent chest pain. A diagnosis of myocardial infarction with non-obstructive coronary arteries secondary to coronary artery vasospasm was determined with the use of multimodality imaging cardiac imaging.

## Introduction

The ubiquity of coronary angiography in the early clinical management of acute myocardial infarction (AMI) has increased the recognition of patients with evidence of myocardial infarction with non-obstructive coronary arteries (MINOCA). MINOCA is defined as AMI in the absence of obstructive coronary artery disease (CAD) (≥50% stenosis) in any coronary artery [1]. Patients with MINOCA are frequently young, non-White females with fewer traditional risk factors compared with those with AMI caused by obstructive CAD [2]. However, MINOCA is a challenging clinical diagnosis and its management remains elusive because the underlying etiology is not immediately apparent. MINOCA accounts for less than 10% of all patients diagnosed with AMI, and carries a 5% risk of mortality at 12 months [3]. Compared with patients with AMI due to obstructive coronaries, those with MINOCA are less likely to have dyslipidemia; however, other cardiovascular risk factors are similar between these two groups [3].

## Case presentation

A female in her early 60s with a past medical history of hypertension, type 2 diabetes mellitus, and recent coronavirus disease 2019 (COVID-19) infection presented with a 24-hour history of intermittent chest pain at rest. The patient was brought to the emergency room by ambulance after experiencing 24 hours of intermittent chest pain (level 6-7/10) that was localized to the midsternal/substernal area at rest. The pain was substernal, non-radiating, and sharp, lasting for 45 minutes. In addition, the pain was associated with nausea, dizziness, and shortness of breath. The patient took aspirin 81 mg and had relief of symptoms. The patient did not have chest pain at admission. Physical examination was notable for a thin woman in no acute distress. Body temperature was 98.6 F˚, pulse 74 bpm, blood pressure 134/77, respiratory rate 16 breaths per minute, and oxygen saturation 95% on room air.

The patient was discharged from our hospital one week prior after being admitted for similar complaints. During that admission, she was found to have a non-ST elevation myocardial infarct (NSTEMI). Coronary angiography was normal. A transthoracic echocardiogram (TTE) revealed normal left ventricular size and function. The workup for hypercoagulability was negative. The patient was discharged home to follow up as an outpatient on aspirin 81 mg daily, simvastatin 40 mg daily, metoprolol succinate 25 mg daily, isosorbide mononitrate 60 mg daily, and amlodipine 5 mg daily.

**Figure 1:**
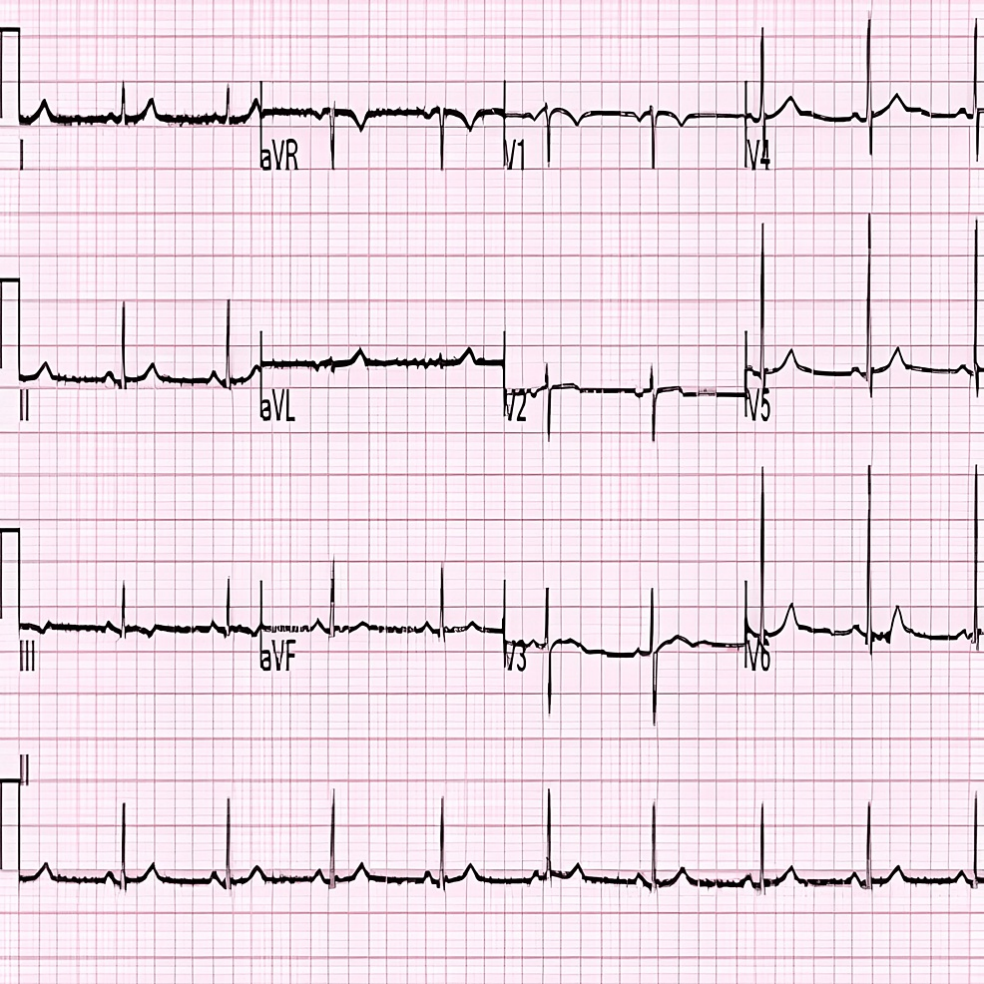
Admission ECG showing sinus rhythm without ischemic changes.

Electrocardiogram in the current presentation demonstrated sinus rhythm, with no ischemic ST/T abnormalities, which was unchanged from the previous admission (Figure 1). Initial troponin I was 0.09 ng/mL and peaked at 0.73 ng/mL (normal ≤0.01 ng/mL). Her labs were otherwise unremarkable.

**Figure 2:**
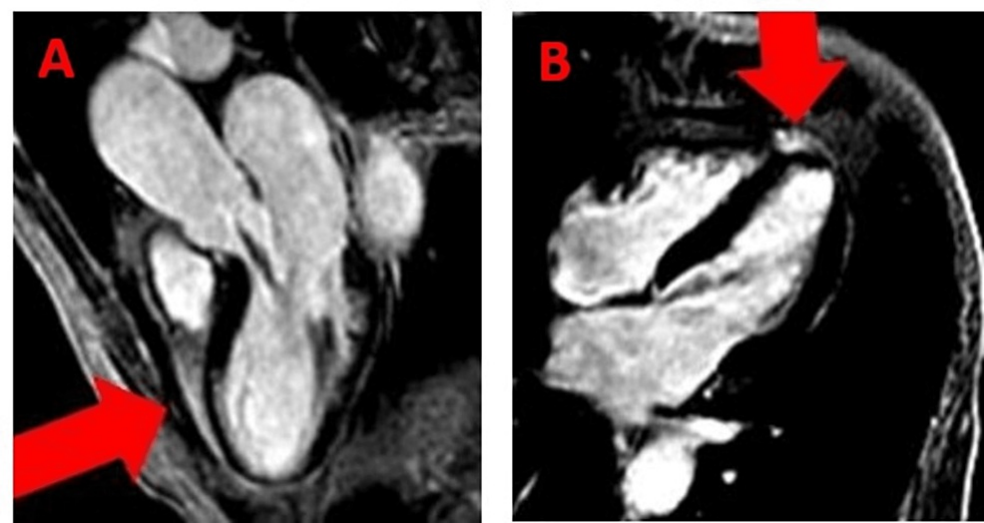
CMR with LGE showed transmural hyperenhancement in the septal wall consistent with acute myocardial infarction (red arrow) (A), and in the apical inferior wall consistent with acute myocardial infarction (red arrow) (B). CMR: cardiac magnetic resonance; LGE: late gadolinium enhancement

Based on the patient’s clinical presentation including recent COVID-19 infection, the patient was managed for possible NSTEMI, pericarditis, or myocarditis with ibuprofen 650 mg three times daily, colchicine 0.6 mg twice daily, rosuvastatin 40 mg nightly, and heparin drip per protocol. The patient did not receive sublingual nitroglycerin because she did not have chest pain in the emergency department. Given her recent admission for NSTEMI with evidence of non-obstructive CAD, a left heart catheterization was initially deferred and a cardiac magnetic resonance (CMR) was obtained. CMR demonstrated severely hypokinetic apical and distal inferior wall with a small focus of subendocardial scar involving the distal septum (Figure 2). There was no evidence of myocarditis or pericarditis by CMR.

**Figure 3:**
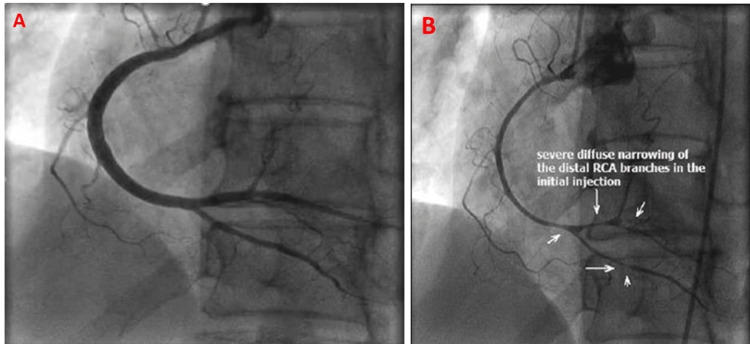
Coronary angiography showing patient’s normal RCA (A), and severe diffuse narrowing of RCA demonstrating vasospasm (white arrows) (B). RCA: right coronary artery

**Figure 4:**
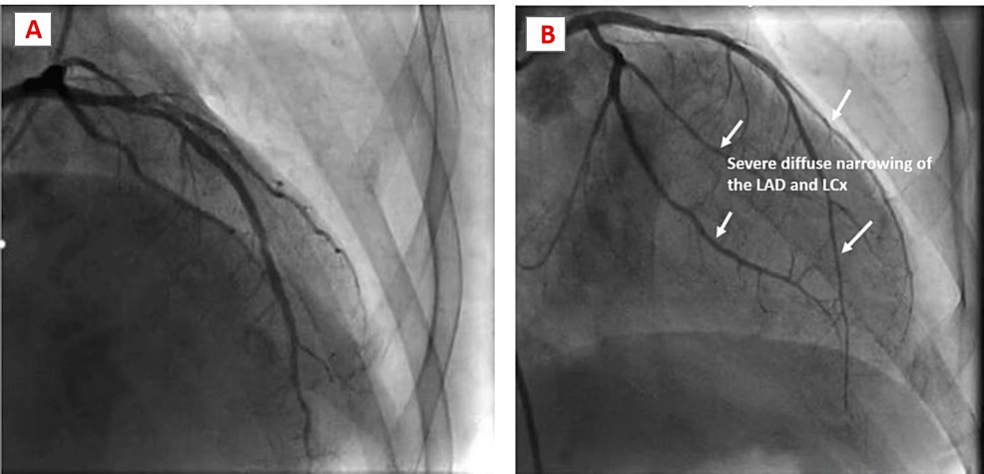
Coronary angiography demonstrating patient’s normal left coronary artery with its distributaries (A), and severe diffuse narrowing of LAD and LCx arteries with vasospasm (white arrows) (B). LAD: left anterior descending; LCx: left circumflex

Given these CMR findings of segmental wall motion abnormalities with associated scar, repeat invasive coronary angiography was performed. The angiography demonstrated severe vasospasm of the entire right coronary artery (Figure 3). In addition, the angiography demonstrated severe extensive vasospasms on the left anterior descending artery and the left circumflex artery (Figure 4), which resolved with intracoronary nitroglycerin. These discoveries were not a result of periprocedure invasive coronary reactivity testing using vasoactive agents such as acetylcholine but rather spontaneous. The patient experienced some mild to moderate chest pain during the procedure, which resolved with intracoronary nitroglycerin.

Given the absence of angiographic evidence of obstructive CAD, this case fits the diagnostic criteria of MINOCA, which is characterized by the diagnosis of AMI without evidence of obstructive CAD on angiography and no alternative clinically apparent cause for presentation. More specifically, the patient’s acute presentation was secondary to coronary artery vasospasm with possible contribution from plaque disruption of her known mild left anterior descending artery (LAD) lesions, resulting in scar/infarct on CMR in this coronary territory.

Isosorbide mononitrate 60 mg daily was added as she had been intolerant of amlodipine in the past. Remaining home medications were continued. Colchicine was discontinued. After initiation of isosorbide mononitrate for the management of coronary vasospasm, the patient’s exertional chest pain resolved. The patient was able to tolerate ambulation and a normal diet. She was discharged home with continued monitoring as an outpatient.

A review of outpatient records reveals that losartan was discontinued due to multiple episodes of low blood pressure and lightheadedness. At the six-month follow-up, the patient reported responding well with improvement of symptoms.

## Discussion

The etiologies of MINOCA are either atherosclerotic or non-atherosclerotic. Myocarditis or Takotsubo cardiomyopathy are not included in the entity of MINOCA, but must be evaluated for and excluded as they can present in a similar manner [3]. Moreover, it is imperative to exclude systemic conditions such as sepsis or pulmonary embolism, that cause supply-demand mismatch resulting in troponin elevation.

Coronary spasm is often an important etiology of angina that may progress to myocardial infarction or sudden death [4]. However, because vascular spasms are transient, patient's electrocardiograms and coronary angiograms may often appear normal at the time of evaluation. In such cases, an intracoronary stimulation test should be considered to diagnose coronary artery spasm, as diagnosis in turn alters treatment with initiation of calcium channel blockers or long acting nitrates [5].

Coronary spasms can result from catheter-induced. Catheter-induced coronary artery spasm can occur during routine coronary angiography, invariably in the right coronary artery (RCA) [6]. It usually occurs proximally to the catheter tip and is often asymptomatic. On the other hand, variant angina spasm, which we believe is the case for our patient, is usually multivessel and not confined to the RCA (in our case RCA, LAD, left circumflex artery (LCx)) [6]. Moreover, the pattern of narrowing is generally diffuse and extensive within an artery, as demonstrated in our case [6]. 

Initially, the work-up for MINOCA includes echocardiography and left ventriculography, which may provide information about the ejection fraction and wall motion abnormalities. Coronary angiography remains the gold standard to assess coronary vessel status and diagnose acute coronary syndrome. However, coronary angiography does not evaluate for coronary reactivity. Consequently, it is highly likely to miss identifying the etiology behind the myocardial injury in these patients [7]. Therefore, diagnostic tests such as invasive coronary reactivity testing (CRT) also known as spasm provocation test can be useful. CRT is an angiographic procedure that evaluates the coronary artery microcirculation and how the small blood vessels respond to different medications. It allows for the diagnosis and more specific treatment of patients with vasospastic disease. CRT is often done in outpatient settings. During CRT, vasoactive medications such as nitrates or caffeine are withheld for a period of time. In our case, the patient did not undergo CRT outpatient due to the acuteness of her symptoms and was brought immediately to the cardiac cath lab.

CMR imaging has emerged as an important diagnostic tool in the initial investigation of MINOCA, especially when a clear cause is not found, largely due to its ability to characterize the myocardium [6]. CMR is recommended within seven days of presentation because delayed imaging can sometimes result in some features no longer being evident [6]. CMR imaging using late gadolinium enhancement (LGE) and cardiac T1 and T2 mapping provide the additive role in the evaluation of myocardial involvement and cardiac function [8]. CMR imaging studies on MINOCA patients can also detect cardiac abnormalities such as hypertrophic cardiomyopathy, dilated cardiomyopathy, pericarditis, and amyloidosis.

The management of MINOCA is challenging because patients may manifest a different cardiovascular risk profile than obstructive CAD patients. Currently, the therapeutic approach is based on targeted therapies once an underlying diagnosis is identified including the targeting of known cardiovascular risk factors and prescribing angiotensin-converting enzyme inhibitors and beta-blockers to alleviate symptoms [9]. MINOCA caused by coronary spasm is treated with calcium-channel blockers (CCB) or nitrates [7]. Most importantly, lifestyle changes such as involvement in sports, alcohol and smoking cessation, weight loss, high-fiber diet with increased consumption of fruits and vegetables have shown benefits for the prognosis of MINOCA patients [10].

MINOCA is not a benign entity even though the long-term prognosis is typically thought to be better compared to patients with myocardial infarction due to obstructive CAD. For example, patients with MINOCA had similar hospital lengths of stay and similar incidences of cardiac arrest, reduced ejection fraction, and heart failure compared to patients with MI from CAD [11, 12]. Ultimately, many MINOCA studies are limited by their observational design and relatively small populations, further emphasizing the need for future large randomized trials to better understand this entity and optimize the care of these patients.

## Conclusions

MINOCA is a challenging clinical diagnosis. MINOCA may present with angina pectoris; however, because vascular spasms are transient, electrocardiography and coronary angiography may often appear normal at the time of evaluation. CMR imaging is a key diagnostic tool in the preliminary investigation of MINOCA when a clear cause is not found. Ultimately, many MINOCA studies are limited by their observational design and relatively small populations, further emphasizing the need for future large randomized trials to better understand this entity and optimize the care of these patients.
